# Supplementary material for: Co-Production of Poly(3-hydroxybutyrate) and Gluconic Acid from Glucose by Halomonas elongata
Source: Bioengineering (Basel). 2023 May 25;10(6):643. doi: 10.3390/bioengineering10060643 (PMC10294990; doi:10.3390/bioengineering10060643)
Supplement: Supplementary file 1 [file bioengineering-10-00643-s001.zip › bioengineering-2362569-supplementary.pdf]

Article

Co-Production of Poly(3-Hydroxybutyrate) and Gluconic Acid from Glucose by *Halomonas elongata*

Tânia Leandro [1,2], M. Conceição Oliveira [3], M. Manuela R. da Fonseca [1,2] and M. Teresa Cesário [1,2,*]

1 IBB-Institute for Bioengineering and Biosciences, Bioengineering Department, Instituto Superior Técnico, Universidade de Lisboa, Av. Rovisco Pais, 1049-001 Lisboa, Portugal; tania.leandro@tecnico.ulisboa.pt (T.L.); manuela.fonseca@tecnico.ulisboa.pt (M.M.R.d.F.)
2 Associate Laboratory i4HB—Institute for Health and Bioeconomy, Instituto Superior Técnico, Universidade de Lisboa—Portugal, 1049-001 Lisbon, Portugal
3 Centro de Química Estrutural, Institute of Molecular Sciences, Instituto Superior Técnico, Universidade de Lisboa, Av. Rovisco Pais, 1049-001 Lisboa, Portugal; conceicao.oliveira@tecnico.ulisboa.pt
* Correspondence: teresa.cesario@tecnico.ulisboa.pt

Supplementary Materials

Co-Production of Poly(3-Hydroxybutyrate) and Gluconic Acid from Glucose by *Halomonas elongata*

Tânia Leandro [1,2], M. Conceição Oliveira [3], M. Manuela R. da Fonseca [1,2] and M. Teresa Cesário [1,2,*]

1 IBB-Institute for Bioengineering and Biosciences, Bioengineering Department, Instituto Superior Técnico, Universidade de Lisboa, Av. Rovisco Pais, 1049-001 Lisboa, Portugal; tania.leandro@tecnico.ulisboa.pt (T.L.); manuela.fonseca@tecnico.ulisboa.pt (M.M.R.d.F.)
2 Associate Laboratory i4HB—Institute for Health and Bioeconomy, Instituto Superior Técnico, Universidade de Lisboa—Portugal, Lisbon, Portugal
3 Centro de Química Estrutural, Institute of Molecular Sciences, Instituto Superior Técnico, Universidade de Lisboa, Av. Rovisco Pais, 1049-001 Lisboa, Portugal; conceicao.oliveira@tecnico.ulisboa.pt
* Correspondence: teresa.cesario@tecnico.ulisboa.pt

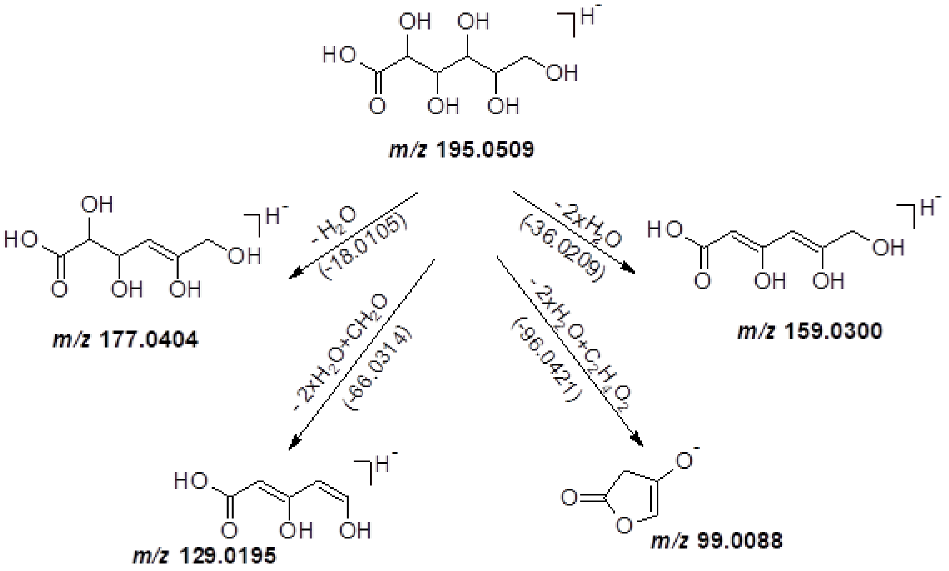

Figure S1. Proposed fragmentation pattern for the deprotonated molecule of gluconic acid *m/z* 195.0509 $[C_6H_{11}O_7]^-$.

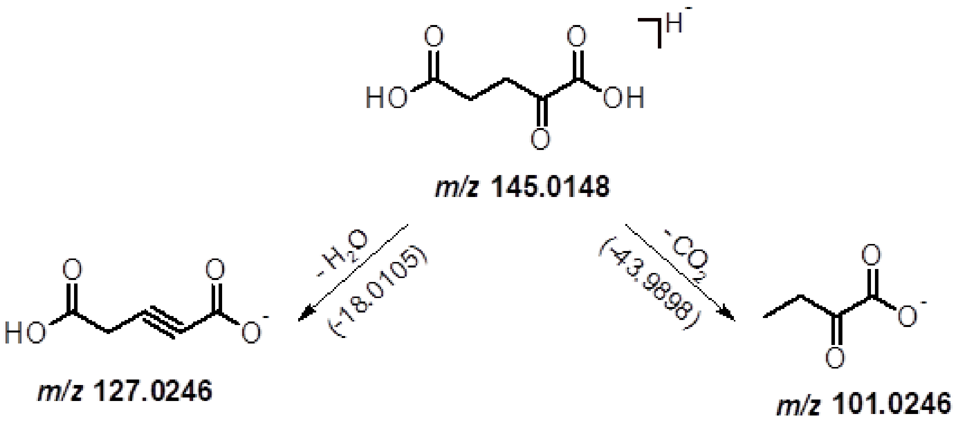

Figure S2. Proposed fragmentation pattern for the deprotonated molecule 2-oxoglutaric acid *m*/*z* 145.0148 $[C_5H_5O_5]^-$.
